# Affinity Chromatography With Selective Nucleic Acid Hybridization: Direct Coupling With Size Exclusion Chromatography

**DOI:** 10.1002/jssc.70555

**Published:** 2026-10-17

**Authors:** Martin Gilar, Brittney Racioppo, Beatrice Muriithi, Kevin Wyndham

**Affiliations:** ^1^ Waters Corporation Milford Massachusetts USA

**Keywords:** affinity chromatography, genotyping, hybridization, messenger RNA, poly(A) tail

## Abstract

Affinity liquid chromatography (aLC) with immobilized oligonucleotides provides high selectivity for nucleic acid targets through on‐column hybridization. We describe the preparation and evaluation of prototype 2.1 mm × 20 mm affinity columns packed with a 3.5 µm nonporous polystyrene/divinylbenzene sorbent covalently modified with streptavidin. Two different affinity columns were prepared by immobilizing biotinylated oligonucleotides on‐column via biotin‐streptavidin bond: (i) biotinylated 25‐nt oligodeoxythymidine, dT25 or (ii) biotinylated 21 nt TP53 oligonucleotide. dT25 column was used for poly(A) denylated messenger RNA (mRNA) and adenosine oligonucleotides variants analysis. The TP53 column was applied to single‐nucleotide polymorphism analysis. Nucleic acid binding was performed in 0.1 M sodium phosphate buffer (pH 7.4); retained species were eluted with water or 10% acetonitrile solution in water. We demonstrate that dT25 column selectively retains poly(A)‐tailed EPO, EGFP, and Cas9 mRNAs, while tailless mRNA is unretained. dT25 column resolved dA14–dA25 oligoadenosine length variants and dA25 variants bearing cytidine substitutions. The TP53 column baseline‐separated a fully complementary 21‐nt sequence from single‐nucleotide mismatches. Direct coupling of the dT25 column with a 250 Å, 1.7 µm size exclusion chromatography (SEC) column enabled improved poly(A) tail sizing after RNase T1 digestion of intact mRNA; mRNA recovery exceeded 98%, and carryover remained below 0.1% after cleanup.

## Graphical abstract

Affinity LC column with immobilized biotinylated oligonucleotide on streptavidin sorbent can separate DNA (RNA) sequence variants.

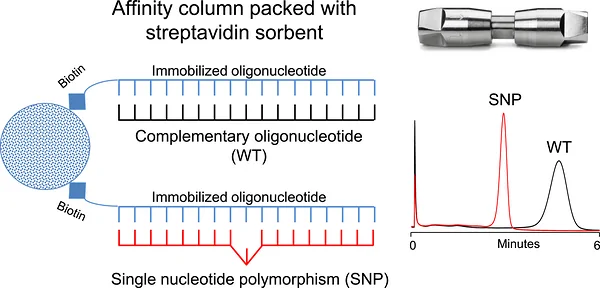

## 1 Introduction

Affinity chromatography is a highly selective liquid chromatography (LC) technique that separates target analyte from other components of complex matrices or biological mixtures. The technique was pioneered in the late 1960s and has since become a powerful tool for purification, isolation, characterization, and analysis of biological molecules [1]. Furthermore, affinity chromatography is a tool to study binding kinetics, thermodynamics, and molecular recognition processes, making it a suitable platform for target protein—drug interaction studies [2, 3].

In contrast to conventional chromatographic methods, for example, reversed‐phase LC (RP LC), which can retain and resolve wide range of molecules according to their moderate Retention On Sorbent due to general analyte hydrophobicity, affinity liquid chromatography (aLC) relies on strong retention via specific molecular recognition between analyte and ligand, such as antigen–antibody, or nucleic acid‐nucleic acid via sequence‐specific hybridization [4, 5, 6].

Due to the high selectivity of affinity ligand immobilized on chromatographic sorbent, aLC separation typically produces two fractions: (i) fraction containing unretained sample components and (ii) fraction containing strongly retained target analyte or class of compounds sharing the epitope with high affinity toward the immobilized ligand. The second fraction is typically eluted with a step gradient of solvent that counteracts the affinity interaction and releases the target analyte from the column. This aLC “bind‐and‐elute” mechanism is used for both analytical applications and purification of target molecules [7, 8].

Examples of immobilized ligands on the chromatographic sorbent include antibodies (mAbs), class of proteins with affinity toward immunoglobulins (protein A, G, and L), peptides, aptamers, nanobodies, lectins, and others [1, 3, 7]. Affinity ligands are often biopolymers providing specific molecular recognition. The disadvantage of such affinity ligands is that they can lose their three‐dimensional structure (via irreversible denaturation) and therefore their activity if aggressive elution conditions are used. Common aLC applications include mAbs purification on protein A or protein G columns, and purification of messenger ribonucleic acid (mRNA) with deoxythymidine columns [9, 10, 11, 12]. The high specificity of aLC enables efficient purification in a single chromatographic step, reducing processing time and improving target product quality. Furthermore, aLC is widely used in biopharmaceutical manufacturing and downstream processing [13].

Affinity chromatography with immobilized oligonucleotides uses affinity interaction principles different from those of protein or peptide‐based columns. The selectivity is achieved via on‐column hybridization of nucleic target analytes with complementary immobilized oligonucleotides. Immobilized oligonucleotide ligand (typically 15–25 nt long) provides high selectivity for isolation of target nucleic acids [14, 15, 16]. Nucleic acid hybridization in aLC offers similar or greater hybridization selectivity as hybridization arrays [17] or sample extraction methods using magnetic beads with immobilized complementary sequence [12, 18, 19]. The latter example, magnetic beads are often used for selective extraction of trace amounts of therapeutic oligonucleotides from biological samples, providing improved sensitivity and specificity for liquid chromatography‐mass spectrometry (LC‐MS) analytical workflows [16].

Despite its high selectivity, affinity chromatography has several limitations. Preparation of affinity ligands can be costly and time‐consuming. Affinity LC sorbents are generally more expensive than conventional chromatographic media, increasing operational costs. Harsh immobilization or elution conditions can deactivate (denature) the chosen ligand, reducing the column lifetime. Also, nonspecific analyte adsorption can compromise affinity LC selectivity.

In a previous study, we described on‐column immobilization of biotinylated affinity ligands on streptavidin columns and investigated the resulting aLC column performance and stability [20]. In the current work, we explore oligonucleotide affinity LC columns. The goal was to investigate the ability of aLC to retain and separate nucleic acids via hybridization with a specific immobilized oligonucleotide. We investigated aLC loading and elution conditions and the selective retention of target nucleic acids. dT25 oligodeoxythymidine column was evaluated with 16–25 nt long oligodeoxyadenosine sequences and their closely related variants. The goal was to demonstrate the applicability of aLC to single nucleotide polymorphism genotyping or isolation of sequence variants. Finally, we explored the use of a tandem dT25/SEC method with the aLC column connected in series with size exclusion chromatography column. This method enabled improved poly(A) tail analysis of digested mRNA.

## 2 Methods and Materials

### 2.1 Chemicals

Sodium phosphate monobasic (≥ 99.0%, catalog no. S8282), and sodium phosphate dibasic (≥ 99.0%, catalog no. S7907) were purchased from Sigma‐Aldrich (St. Louis, MO, USA). Ammonium acetate (> 99.995%) was purchased from Millipore‐Sigma (Burlington, MA, USA). RNase T1 endoribonuclease (catalog no. EN0541) and D‐biotin (catalog no. BP232‐1) were purchased from Thermo Fisher Scientific (Waltham, MA, USA). All mobile phase buffers were prepared using Milli‐Q water and filtered through a 0.2‐µm filter (Cat. No. FB12566508) purchased from Fisher Scientific (Hampton, NH, USA). LC‐MS grade acetonitrile and MS‐grade acetic acid were purchased from Fisher Scientific (Hampton, NH, USA). Oligonucleotides were purchased from Integrated DNA Technologies (Coralville, IA, USA); the sequences and modifications are listed in Table 1. Among these are 5’‐biotinylated dT25 oligodeoxythymidine (25 nucleotides long, HPLC purified), dT150 oligodeoxythymidine (150 nucleotides long, HPLC purified), dA16, dA17, dA18, dA19, dA22, and dA25 oligodeoxyadenosines (crude). The 25 nt long oligodeoxyadenosine sequences variants (see Table 1) had one, two, or three nucleotides replaced with cytidine nucleotides. Synthetic crude ribonucleotide adenosines rA30, rA40, rA60, rA80 and rA120 samples were used for size exclusion column calibration without purification. Biotinylated TP53 ligand and its 21 nt oligonucleotide semicomplementary targets (21G WT, 21T, 21A, 21C) sequences are listed in Table 1. The synthetic DNA or RNA oligonucleotides were not chemically modified (apart from 5’‐biotinylation as indicated). N1‐methyl‐pseudouridine‐modified M6 EPO mRNA, part no. L‐8109 (859 nt), M6 EGFP mRNA, part no. L‐8101 (997 nt), M6 Fluc mRNA, part no. L‐8102 (1922 nt), and M6 Cas9 mRNA, part no. L‐8106 (4522 nt), were purchased from TriLink BioTechnologies (San Diego, CA, USA). NIST Fluc nRNA, part no. RGTM 10202, was purchased from National Institute of Standards and Technology (NIST, Gaithersburg, MD, USA). Tailless 2180 nt mRNA was obtained from Kactus Bio (Waltham, MA, USA).

**TABLE 1:** Oligonucleotides used in this study.

Oligonucleotide description	Oligonucleotide length (nt)	Sequence (5’–3’)^a^
Biot dT25	25	Biotin‐TTT TTT TTT TTT TTT TTT TTT TTT T
dA25	25	AAA AAA AAA AAA AAA AAA AAA AAA A
dA22	22	AAA AAA AAA AAA AAA AAA AAA A
dA19	19	AAA AAA AAA AAA AAA AAA A
dA18	18	AAA AAA AAA AAA AAA AAA
dA17	17	AAA AAA AAA AAA AAA A A
dA16	16	AAA AAA AAA AAA AAA A
5'C	25	AA **C** AAA AAA AAA AAA AAA AAA AAA A
3'C	25	AAA AAA AAA AAA AAA AAA AAA A **C** A A
Mid C	25	AAA AAA AAA AAA **C** AA AAA AAA AAA A
Mid 2C	25	AAA AAA **C** AA AAA **C** AA AAA AAA AAA A
Cent 3C	25	AAA AAA AAA AA **C CC** A AAA AAA AAA A
Mid 3C	25	AAA AAA **C** AA AAA **C** AA AAA **C** AA AAA A
Biot *TP53*	21	Biotin‐AAC TGT TAC ACA TGT AGT GTA
21G WT	21	TAC ACT ACA T **G** T GTA ACA GTT
21T	21	TAC ACT ACA T **T** T GTA ACA GTT
21A	21	TAC ACT ACA T **A** T GTA ACA GTT
21C	21	TAC ACT ACA T **C** T GTA ACA GTT
dT150	150	T(T)_148_T
rA30	30	A(A)_28_A
rA40	40	A(A)_38_A
rA60	60	A(A)_58_A
rA80	80	A(A)_78_A
rA120	120	A(A)_118_A

^a^ Bold underlined font indicates the oligonucleotide sequence positions varied in the experiment.

### 2.2 Instrumentation and Columns

Chromatographic experiments were performed using ACQUITY Premier System consisting of a quaternary solvent manager (QSM), Flow Through Needle Sample Manager (FTN SM), column heater manager (CM‐A) with enabled active preheater (APH), and ACQUITY Photodiode Array (PDA) Ultraviolet Detector with a 5 mm titanium flow cell. Genotyping affinity LC experiments were performed with ACQUITY TUV Detector with 10 mm analytical flow cell. Data were acquired and processed with Empower Software 3.0 from Waters Corporation (Milford, MA).

Ultra‐high‐performance liquid chromatography (UHPLC) affinity LC column prototypes were packed in house. The 2.1 mm × 20 mm (i.d. × length) Waters MaxPeak Premier Column Hardware was packed in‐house with 3.5 µm nonporous polystyrene/DVB hydrophilic particles conjugated with streptavidin.

SEC analysis of intact and RNase T1‐digested mRNA (digestion releases poly(A) tail, typically 30–150 nt long) was performed with ACQUITY Premier Protein SEC Column, 250Å, 1.7 µm, 4.6 mm ×150 mm, obtained from Waters Corporation. The affinity LC, SEC, and aLC x SEC tandem methods are described in the following sections.

### 2.3 Affinity Ligand On‐Column Bonding

Note that 2.1 mm × 20 mm columns packed with streptavidin sorbent enable on‐column coupling with biotinylated ligands (via streptavidin‐biotin bond). The on‐column coupling was performed as described in a recent report [20]. Briefly, a series of injections of 5’ biotinylated oligonucleotide ligand solution were performed using UHPLC system in 0.1 M sodium phosphate buffer, pH 7.4 (buffer was prepared by dissolving 5.75 g Na_2_HPO_4_ and 1.14 g NaH_2_PO_4_ in 500 mL of deionized Milli‐Q water). The ligand on‐column coupling was performed at a flow rate 0.15 mL/min and 25°C. UV signal at the column outlet was monitored with UV detector at UV 260 nm or UV 290 nm. Significant (full) oligonucleotide breakthrough detected as UV signal indicated that the column was saturated with the ligand.

Remaining streptavidin active sites present on the sorbent (inaccessible to oligonucleotide bonding due to steric hindrances) were endcapped with subsequent injections of biotin solution until the biotin breakthrough was detected at UV 210 nm [20]. The endcapping procedure was implemented to ensure that all active streptavidin binding sites were saturated, and no biotinylated ligand could be inadvertently immobilized throughout later column use. Two affinity LC columns prepared for this study were as follows: (i) dT25 column with 5’ biotinylated 25 nt oligodeoxythymidine (14 nmol of Biot dT25 ligand was immobilized on 2.1 mm × 20 mm column) [20] and (ii) TP53 column prepared using 5’ biotinylated 21 nt TP53 oligonucleotide (Biot TP53 sequence see Table 1; 13 nmol of Biot TP53 ligand was immobilized on 2.1 mm × 20 mm column). Approximately thirty 2 µL injections (0.25 nmol/µL of ligand) were sufficient to saturate the streptavidin 2.1 mm × 20 mm column with biotinylated oligonucleotides. For affinity column preparation, see Figure S1 and a recent report [20]. Bonded affinity columns were washed with five blank gradients (Section 2.4) prior to use. Columns were stored in 10% acetonitrile at 10°C between experiments.

### 2.4 Affinity LC Methods: Target Nucleic Acid Capture and Elution Conditions

The dT25 column was used for analysis of poly(A)‐tailed mRNA and oligodeoxyadenosine variants, while TP53 column was used for genotyping applications. Both columns were used with 0.1 M of sodium phosphate buffer, pH 7.4 (181 mM of [Na^+^] concentration) for target sequence trapping. A 0.1 M ammonium acetate (pH 6.8) as hybridization buffer was also used. Higher buffer concentration enhances hybridization strength (greater retention on column), while lower buffer concentrations yield weaker retention. Nucleic acids captured on aLC columns can be eluted by applying denaturing conditions that disrupt the hydrogen bonds and base‐stacking interactions between duplex and convert it into single‐stranded species. Denaturing conditions include elevated column temperature, high mobile phase pH, addition of organic solvents, or mobile phase with low ionic strength. The most practical elution approach in aLC is a gradient of water or 10% acetonitrile in water. We used a step gradient from 100 % A to 100 % B initiated at 0.01 min. A 100% B conditions were held till 2.5 min; at 2.51 min, a second step gradient from 100% B back to 100% A was performed to equilibrate the column at 100% A until 5 min. The flow rate was 0.4 mL/min, column temperature at 25°C, and UV detection wavelength 260 nm. For separation of DNA sequence variants, we used linear gradients; the conditions are specified in figure captions. In a separate study, we investigated the effect of mobile phase composition on siRNA duplex stability [21]. Elevated temperature and organic solvents will result in faster elution of target nucleic acids; however, temperatures above 35°C and high concentrations of organic solvents (> 10%) in the mobile phase will result in shorter aLC column lifetime. dT25 column stability was investigated in a recent study [20].

### 2.5 RNase T1 mRNA Digestion Followed With SEC Analysis

mRNA samples were digested with RNase T1 using the following protocol: 10 µL of the mRNA (0.5–1 mg/mL) was mixed with 30 µL of sodium phosphate buffer, pH 7.4. 1µL (1 unit) of RNase T1 enzyme was added to the sample, and the digestion was carried out at 37°C for 30–60 min.

### 2.6 Tandem aLC/SEC Method

Prototype 2.1 mm × 20 mm dT25 column was serially connected to ACQUITY Premier Protein SEC Column, 250Å, 1.7 µm, 4.6 mm ×150 mm using 0.15 mm × 50 mm stainless steel tubing. Mobile phase was 0.1 M sodium phosphate buffer, pH 7.4, column temperature 25°C. Injected mRNA digest components that were unretained on dT25 column (flow through Fraction 1, Fr 1) proceed to SEC column where they were directly resolved and recorded at UV 260 nm as a first chromatogram. After the separation of Fraction 1 was finished, the retained species (Fraction 2, Fr 2) were released from dT25 column by 25 µL injection of water or 10% acetonitrile in water. The “injection‐elution” was performed by the UHPLC system. This elution protocol generates a sharp peak of poly(A)‐retained species (< 10 µL peak volume at 50% height). Injection‐elution approach was chosen to minimize peak broadening due to dT25 column prior to SEC analysis and to minimize running mobile phase composition disruption. SEC separation of second fraction was recorded as a second chromatogram. After the dT25/SEC analysis, the column tandem was washed with four 25 µL injections of 10% acetonitrile solution spaced by 1 min to remove all mRNA residues from the dT25 column and minimize sample carryover into subsequent analyses.

## 3 Results and Discussion

### 3.1 dT25 Column Application to mRNA Analysis

Affinity dTn columns are used in the industry for purification of mRNA [9, 10, 11, 22, 23]. mRNA is retained via poly(A) tail (typically 100–130 nt long) interactions with the immobilized oligodeoxythymidine ligand. The prototype 2.1 mm × 20 mm dT25 column is too small for preparative application; it was developed for mRNA analytical purposes. Figure 1 shows the proof of concept for analysis of three purified mRNA samples: a 2180 nt KH20 mRNA without poly(A) tail (Figure 1A), 997 nt EGFP mRNA containing poly(A) tail (Figure 1B), and 4522 nt Cas9 mRNA containing poly(A) tail (Figure 1C). Note that 2.5 µg of mRNA was injected on dT25 column and eluted with gradient described in Section 2.4.

**FIGURE 1:**
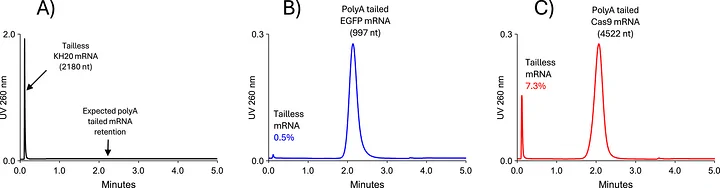
Analysis of mRNA with dT25 affinity column. (A) Tailless KH20 mRNA is not retained on column. Arrow at 2.1 min denotes the expected position of poly(A) tailed mRNA; no peak was present. (B) Poly(A) tailed EGFP mRNA and (C) poly(A) tailed Cas9 eluted near 2.1 min time. Only 0.5% of tailless mRNA was observed in EGFP mRNA sample and 7.3% in Cas9 mRNA sample; see chromatograms (B) and (C), respectively. For the gradient method, see Section 2.4.

Figure 1 shows that tailless KH20 mRNA is not retained on column; it elutes as a sharp breakthrough peak at 0.1 min. Both EGFP and Cas9 mRNAs are retained on dT25 column via their poly(A) tail hybridization with dT25 ligand on column in 0.1 M sodium phosphate buffer. Retained mRNA samples were eluted with gradient water (see peaks at 2.1 min in Figure 1B,C). This experiment confirms that poly(A) tail is required for mRNA retention on dT25 column and that tailless KH20 mRNA is not retained on the column by nonspecific interactions with the sorbent (Figure 1A).

Figure 1B,C show that minor peak at 0.1 min is present in the purchased (purified) mRNA samples. This peak is presumably a tailless version of EGFP mRNA (0.5 % of UV area at 260 nm) and Cas9 mRNA (7.3%). The observed percentage of tailless mRNA (first peak) did not change with the mRNA mass load (0.5–10 µg; data not shown), leading us to believe that this peak is not mRNA breakthrough due to column overload with the sample. The reported capacity of 2.1 mm × 20 mm dT25 column is > 40 µg mRNA (mass load improves for smaller mRNA) [9, 20].

To investigate the matter further, we collected both first and second mRNA peaks and checked for the presence of poly(A) tail in the sample [20]. The results confirmed that dT25 column first (unretained) peak does not contain a poly(A) tail (Figure S2).

### 3.2 dT25 Column Retention and Selectivity for Adenosine Oligonucleotides

Several dTn affinity sorbents are commercially available for purification of mRNA [11, 22]. The length of immobilized dTn ligand ranges from dT14 for Sera‐Mag magnetic beads, dT18 for CIMmultus monolith columns [10], dT20 for Monomix dT20 columns, to dT25 for Poros (dT)25 sorbents. However, a limited number of studies describing the length of ligand necessary for successful retention of poly(A) structures have been published [10, 14, 15]. We investigated the binding and elution behavior of short dAn oligodeoxyadenosines on 2.1 mm × 20 mm dT25 column using 0.1 M sodium phosphate binding buffer, pH 7.4, linear gradient of 10% acetonitrile, and 35°C column temperature. Figure 2A confirms that even relatively short dAn oligonucleotides can be retained and partially resolved on dT25 column. Shorter than dA10 oligonucleotides eluted in column void time under the separation conditions (dA1–15 nt by‐products of oligonucleotide synthesis are present as minor peaks in the sample). The retention improved for longer A‐T duplexes; Figure 2A suggests that ∼14–25 nt long dAn with the dT25 immobilized ligand ensure useful retention for oligo‐poly(A) species. The retention can be further adjusted with the choice of binding buffer concentration, addition of Mg^2+^ cations, via column temperature, or with addition of organic denaturants to the eluent [14, 15, 21].

**FIGURE 2:**
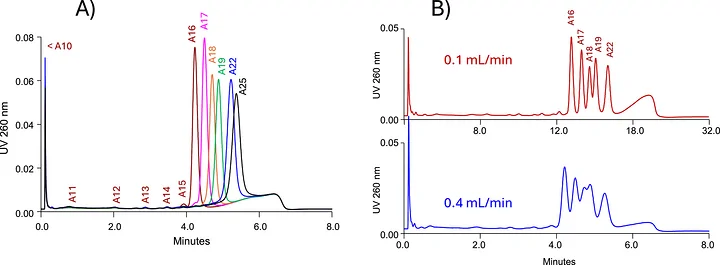
(A) Separation dA10–dA25 oligonucleotides on dT25 column. (B) Separation dA16, dA17, dA18, dA19, and dA22 mixture on dT25 column improves at 0.1 mL/min flow rate. LC conditions: 2.1 mm × 20 mm, 3.5 µm dT25 column; 35°C, mobile phase A: 0.1 M sodium phosphate, pH 7.4; B: 10% acetonitrile. Separations were performed at 0.4 mL/min using linear gradient 0% to 100% B in 5 min, 100% B was held till 5.5 min. Column was re‐equilibrated from 5.51 to 8 min at 100% A. For 0.1 mL/min analysis in panel (B), the gradient time was extended fourfold, that is 0% to 100% B in 20 min, 100% B was held till 22 min, and the column was re‐equilibrated at 100% A for 10 min.

In the next experiment, we investigated separation of 16–22 nt oligodeoxyadenosines. We observed a useful separation of oligonucleotides according to their length sample (Figure 2B). The resolution was further enhanced using fourfold reduced mobile phase flow rate (0.1 mL/min, fourfold extended gradient time to maintain the constant gradient slope) [24, 25]. This behavior indicates that hybridization/melting of A–T duplexes on column is a relatively slow process. Since the column is packed with nonporous sorbent, we do not expect that oligonucleotide slow diffusion (slow mass transfer in the stationary phase pores) is responsible for greater peak broadening observed at 0.4 mL/min flow rate.

Retention principles of dT25 column were further explored with EPO mRNA test sample. Figure S3 shows that EPO mRNA is retained on the affinity column as expected, but the retention is lost upon EPO mRNA poly(A) tail hybridization with dT150 oligonucleotide prior to injection on dT25 column. When the mRNA poly(A) tail hybridizes in solution with dT150, the mRNA sample is no longer able to interact with the immobilized oligonucleotide dT25. This is an important finding that potentially complicates the applications of affinity LC. Molecular biology applications often start with polymerase chain reaction (PCR), which produces double‐stranded amplicons. The amplified PCR products must be converted to single‐stranded species prior to their hybridization on an aLC column with the immobilized complementary ligand.

### 3.3 Affinity LC Columns Discrimination of Sequence Variants

In the next experiment, we investigated whether the affinity column can resolve 25 nt long oligo(A) variants with one, two, or three adenosines replaced with cytidine(s). The oligo(A) variants are listed in Table 1. Figure 3 shows that despite using a short column, affinity LC can resolve single nucleotide variants, including those appearing near the 3’ or 5’ termini. The sequence alteration near the center of oligonucleotide (Mid C) or multiple modifications (Mid 2C, Cent 3C, Mid 3C) destabilize the A–T duplex stability more severely, hence significantly reducing the retention of such oligonucleotide species on affinity column. The experiment presented in Figure 3 suggests that aLC columns with immobilized oligonucleotide ligands are applicable to genotyping or selective enrichment of desirable sequence variants.

**FIGURE 3:**
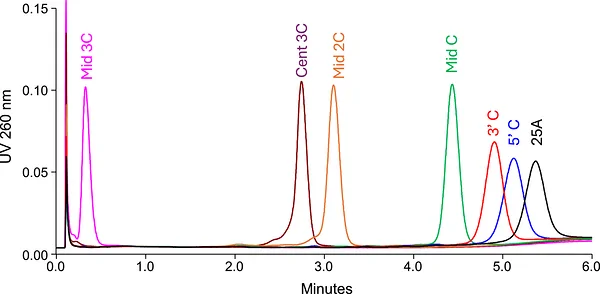
Oligodeoxyadenosine 25 nt sequence variants separated on dT25 affinity LC column. For oligonucleotide sequences see Table 1. LC conditions are the same as in Figure 2A (0.4 mL/min, 35°C, and a 5 min linear gradient).

Encouraged with the results, we prepared TP53 column with immobilized 21 nt oligonucleotide ligand. The ligand sequence is related to *TP53* gene motif (Table 1). Figure 4 illustrates the results for separation of fully complementary 21 nt oligonucleotide (21G WT, where WT means wild‐type) and three oligonucleotides of similar sequence differing by a single nucleotide in the center of sequence (samples 21T, 21A, and 21C where the letter indicates the type of nucleotide). Figure 4 shows that 21T, 21A, and 21C sequence variants are less retained on the column and clearly resolved from the 21G WT oligonucleotide. The experiment in Figure 4 represents proof of concept for affinity LC genotyping. Due to baseline separation of sequence variants, it is in principle possible to characterize homozygote/hetorozygote genotypes and accurately quantify the allele ratios. In addition, we envision that aLC method like the one in Figure 4 may be useful for enrichment of rare variants in target mRNA or DNA motifs prior to next‐generation sequencing (NGS) and enable detection of low‐frequency mutations that are otherwise overlooked.

**FIGURE 4:**
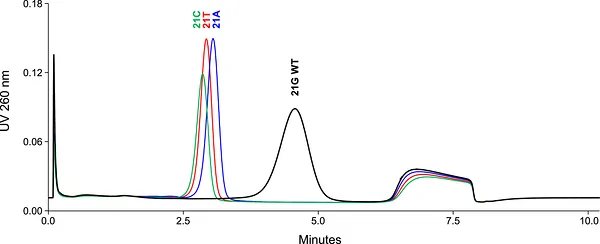
Separation of 21 nt oligonucleotides on 2.1 mm × 20 mm TP53 affinity LC column. 21G WT oligonucleotide sequence is complementary to the immobilized ligand. 21C, 21T, and 21A have single nucleotide miss‐match in the center of sequence (Table 1). LC conditions: 2.1 mm × 20 mm, 3.5 µm dT25 column; 35°C, mobile phase A: 0.1 M ammonium acetate, pH 6.8; B: 50% acetonitrile. Separations were performed at 0.4 mL/min with gradient: 100% A till 0.2 min, 0.2–5.2 min 0% to 100% B, 100% B was held till 7.2 min. Column was re‐equilibrated at 100% A from 7.21 to 10.21 min. Oligonucleotide samples were dissolved in mobile phase A.

### 3.4 Tandem dT25/SEC Method for Advanced mRNA Analysis

In a recent publication, we described SEC method for mRNA poly(A) tail sizing [26]. The method uses enzymatic digestion of mRNA to liberate poly(A) tail using RNase T1 (protocol is described in Section 2.5). The released ∼30–150 poly(A) tail length can be accurately sized using SEC calibration with rA30–rA150 retention standard. While this method is simple and robust, it has some shortcomings highlighted in Figure 5 and Figure S4. (i) Long mRNA (> 4500 nt) digestion will produce dominant signal for short oligonucleotides compared and relatively low signal of released poly(A) tail. (ii) Incomplete mRNA digest can produce fragments (missed cleavages) that interfere with poly(A) tail peak. (iii) The digest of mRNA vaccines [27, 28] or mRNA samples [29] that have poly(A) tail segmented into shorter portions (e.g. 30 nt + 70 nt poly(A) separated by 10 nt spacer, GCAUAUGACU) will produce short poly(A) segments that are more likely to be obscured by other digestion products (see Figure 5C, Fluc mRNA, NIST sample).

**FIGURE 5:**
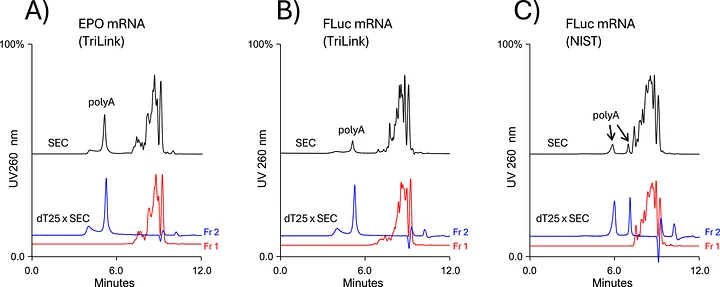
Separation of RNase T1‐digested mRNA using dT25 affinity column (black chromatograms) or dT25/SEC column tandem (red chromatograms representing Fraction 1, and blue chromatograms Fraction 2). (A) Analysis of EPO mRNA, (B) analysis of Flur mRNA, and (C) Fluc mRNA from NIST. Poly(A) tail peaks are labeled in chromatograms. Separation conditions are described in Section 2.6.

The limitations of the method can be resolved by combining dT25 with SEC column as shown in Figure 6A. The role of dT25 column is to enrich and isolate poly(A) tail and remove the digestion background signal (compare SEC and tandem dT25/SEC analyses in Figure 5 and Figure S4). Tandem dT25/SEC method conditions are described in Section 2.6. The method uses isocratic conditions (0.1 M sodium phosphate buffer, pH 7.4) as common binding conditions for dT25 column and SEC mobile phase. Upon injection of mRNA digest, the unretained Fraction 1 passes via dT25 column, proceeds to SEC column as a sharp peak, where it is resolved by the SEC separation mechanism. We chose ACQUITY Premier Protein SEC Column, 250 Å, 1.7 µm, 4.6 mm × 150 mm for this application, because this column is well suited for separation of 30–150 nt long poly(A) species [26]. After the SEC separation is finished, the poly(A) species (Fr 2) still residing on dT25 column are eluted by injection of 25 µL of 10% of acetonitrile. The injected plug of eluent efficiently releases poly(A) species from dT25 in a sharp peak (10 µL peak volume at half peak height). This elution scheme is preferred over eluting dT25 column with a mobile phase gradient, because it results in sharper poly(A) tail peak compared to step gradient elution method (Figure 1) and minimally disrupts the SEC analysis.

**FIGURE 6:**
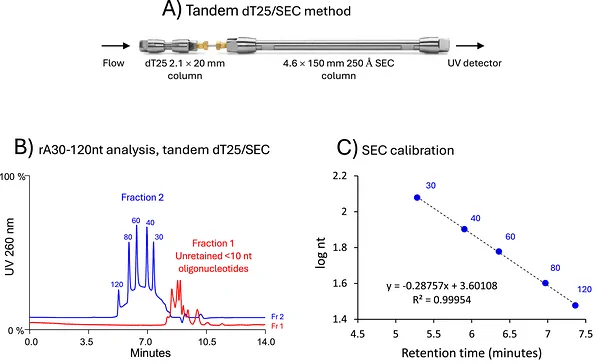
Panel (A) shows a schematic of tandem dT25/SEC column connection. (B) Separation of rA30‐120 nt calibration standard mix. Fraction 1 is dT25 unretained flow through, and Fraction 2 contains dT25 retained oligo(A) species resolved by SEC column. Tandem dT25/SEC method retention times of oligo(A) standards were used to prepare the calibration curve (C) for subsequent mRNA poly(A) tail analysis.

Tandem dT25/SEC method is illustrated in Figure 6 for analysis of rA30‐rA120 calibration mixture. Fraction 1 (dT25 column breakthrough) contains only short rAn oligonucleotides that are not retained on the affinity column. Fraction 2 (Figure 6B) shows well resolved peaks of rAn calibration standards. The bulging baseline is due to rAn failed synthesis products present in oligonucleotide standards rather than due to the peak tailing. Retention times of rA30–rA120 calibration standards were used to prepare the calibration curve shown in Figure 6C. The calibration plot was used to calculate the length of poly(A) for interrogated mRNA samples. Measured poly(A) tail length for EPO, EGFP, Fluc, and Cas9 mRNA samples was between 123 and 124 nt long (Figure S4). This is consistent with the LC MS results in the published report [26]. Please note that SEC method is incapable of resolving N‐1, 2… micro‐heterogeneous species in poly(A) tail, and it is not suitable to characterize poly(A) tail heterogeneity. Instead, SPE method provides an average poly(A) tail length. Figure 5C compares SEC with tandem dT25/SEC method for Fluc mRNA obtained from NIST. The latter approach provides much cleaner peaks of two segments of poly(A) tail and greater confidence in the results (both SEC and dT25/SEC methods yielded identical length estimates for poly(A) segment, *L* = 76.3 and 36.6 nt). These values are 1–3 nt longer than expected values *L* = 75 and 33 nt. This discrepancy is likely due to SEC method averaging the poly(A) tail microheterogeneity into an average poly(A) tail length.

After the calibration shown in Figure 6B, we performed four blank injections using 25 µL injection‐elution. We observed low levels of sample carryover, as shown in Figure 7A). The source of carryover was traced to dT25 column. We attribute the carryover to a limited efficiency of injection‐elution method, since the carryover of dT25 column for step gradient elution method (Figure 1) was below 0.1% of mRNA. We speculate that injection‐elution plug of the strong eluent is diluted with the running mobile phase on dT25 column, and a portion of poly(A) species is readsorbed on aLC column. The carryover was further investigated with EPO mRNA sample in an experiment shown in Figure 7B. Note that 0.5 µg of EPO mRNA sample was injected on dT25/SEC column tandem. The retained sample was eluted from dT25 column with five 25 µL injections of 10% acetonitrile spaced at 1‐min intervals. This experiment enabled us to quantify the injection‐elution efficiency and carryover (Figure 7B). The recovery of mRNA in first 25 µL injection‐elution was greater than 98%, and the additional injections represent the carryover. After the fifth injection‐elution the EPO mRNA signal was 0.1% or less.

**FIGURE 7:**
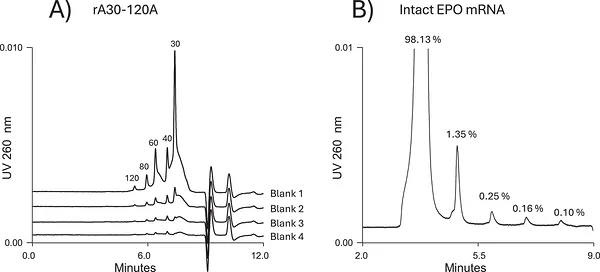
Carryover of tandem dT25/SEC method. (A) Four 25 µL injection‐elution blank runs of tandem dT25/SEC tandem executed after initial rA30‐120 nt calibration run (shown in Figure 6B). (B) Intact EPO mRNA analysis with tandem dT25/SEC method. The first injection‐elution represents *EPO* mRNA recovery, four subsequent injection‐elution blanks represent sample carryover levels.

In a separate experiment, we investigated elution efficiency and carryover levels of the injection‐elution method using a range of injection volumes. We found that carryover persisted even when 50–100 µL injections were employed for elution (data not shown). We believe that low‐level carryover is an inherent drawback of the injection‐elution method (readsorption of analyte on affinity column during injection‐elution; < 0.1% carryover was observed when full gradient elution was used for dT25 column). To correct this shortcoming, we implemented a cleanup protocol after each tandem dT25/SEC analysis using five 25 µL injections of eluent (method similar to the experiment in Figure 7) followed by 12 min isocratic elution to clear carryover from SEC column.

## 4 Concluding Remarks

We demonstrate the utility of affinity‐based liquid chromatography for selective analysis and enrichment of nucleic acid targets using immobilized oligonucleotide ligands. The short dT25 column prototype (2.1 mm × 20 mm) effectively retained oligoadenosine species in mobile phases containing commonly used buffers, including 0.1 M sodium phosphate and ammonium acetate, while providing a good peak shape, high recovery, and low carryover. The retained analytes were eluted with water or 10% acetonitrile in water, enabling quantitation of poly(A)‐tailed mRNA under mildly denaturing conditions. The column exhibited high selectivity, retaining poly(A)‐containing species while excluding tailless mRNA sequences. Further retention studies revealed that oligo(A) species longer than 10 nucleotides were retained via hybridization and resolved on a column according to tail length. Note that 25 nt oligoA sequence variants with one to three adenosine replacements with cytidine were also chromatographically resolved from the parent 25 nt oligoA.

The broader applicability of this approach was demonstrated using an affinity column bearing an immobilized 21 nt oligonucleotide complementary to a TP53 target sequence. This column differentiated a fully complementary sequence from single‐nucleotide variants containing a central mismatch, providing proof of concept that affinity LC can discriminate between wild‐type and SNP‐containing nucleic acid sequences based on hybridization strength. This example illustrates aLC applicability to DNA genotyping.

In addition, coupling the dT25 affinity column with SEC offers a practical strategy for poly(A) tail length analysis of digested mRNA. By selectively enriching the liberated poly(A) tails and removing the remaining tailless digestion mRNA products that interfere with reliable measurements, this tandem workflow improves analytical specificity and supports a robust poly(A) tail length measurement. Presented results establish affinity LC as a versatile platform for selective capture, enrichment, and discrimination of nucleic acid species, with potential applications in mRNA analytics and sequence‐specific nucleic acid characterization.

## Author Contributions

**Martin Gilar**: writing – original draft, supervision, methodology, investigation, formal analysis, conceptualization, visualization. **Brittney Racioppo**: writing – review and editing, investigation, formal analysis. **Beatrice Muriithi**: writing – review and editing, conceptualization, formal analysis. **Kevin Wyndham**: writing – review and editing, conceptualization, project administration.

## Conflicts of Interest

The authors are currently employed by Waters Corporation, the manufacturer of several of the columns that were used in this study.

## Supporting information




**Supporting File**: jssc70555‐sup‐0001‐SuppMat.docx.

## Data Availability

Data will be made available upon request. ACQUITY, Empower, and MaxPreak are trademarks of Waters Corporation or its affiliates. Milli‐Q is a trademark of Merck KGaA. Sera‐Mag is a trademark of Hyclone Laboratories LLC. CIMmultus is a trademark of Sartorius BIA Separations d.o.o. Poros is a trademark of Applied Biosystems LLC. All other trademarks are the property of their respective owners.

## References

[1] E. L. Rodriguez , S. Poddar , S. Iftekhar , et al., “Affinity Chromatography: A Review of Trends and Developments Over the Past 50 Years,” Journal of Chromatography B, Analytical Technologies in the Biomedical and Life Sciences 1157 (2020): 122332. 10.1016/j.jchromb.2020.122332 PMC7584770 32871378

[2] D. S. Hage , J. A. Anguizola , C. Bi , et al., “Pharmaceutical and Biomedical Applications of Affinity Chromatography: Recent Trends and Developments,” Journal of Pharmaceutical and Biomedical Analysis 69 (2012): 93–105. 10.1016/j.jpba.2012.01.004 PMC3346860 22305083

[3] G. Feng , X. Yuan , P. Li , et al., “G Protein‐Coupled Receptor‐In‐Paper, a Versatile Chromatographic Platform to Study Receptor‐Drug Interaction,” Journal of Chromatography A 1637 (2021): 461835. 10.1016/j.chroma.2020.461835 33383241

[4] U. D. Neue , HPLC Columns: Theory, Technology, and Practice (Wiley‐VCH, 1997).

[5] L. Nováková , M. Douša , P. Česla , and J. Urban , Modern HPLC Separations in Theory and Practice (Czech Chromatographic School, 2024).

[6] K. Eguchi , Y. Hamaguchi , Y. Aso , T. Shioiri , M. Ogura , and M. Mitsuhashi , “Oligo(dT)‐Immobilized Pipette Tip: Efficient New Methodology for mRNA Preparation and Direct Gene Amplification,” Clinical Chemistry 44 (1998): 2208–2210. 9761262

[7] E. L. Pfaunmiller , M. L. Paulemond , C. M. Dupper , and D. S. Hage , “Affinity Monolith Chromatography: A Review of Principles and Recent Analytical Applications,” Analytical and Bioanalytical Chemistry 405 (2013): 2133–2145. 10.1007/s00216-012-6568-4 PMC3578177 23187827

[8] Z. Li , E. Rodriguez , S. Azaria , A. Pekarek , and D. S. Hage , “Affinity Monolith Chromatography: A Review of General Principles and Applications,” Electrophoresis 38 (2017): 2837–2850. 10.1002/elps.201700101 PMC5671914 28474739

[9] E. A. Dewar , P. Guterstam , D. Holland , et al., “Improved mRNA Affinity Chromatography Binding Capacity and Throughput Using an Oligo‐dT Immobilized Electrospun Polymer Nanofiber Adsorbent,” Journal of Chromatography A 1717 (2024): 464670. 10.1016/j.chroma.2024.464670 38310705

[10] N. Mencin , D. Štepec , A. Margon , et al., “Development and Scale‐Up of Oligo‐dT Monolithic Chromatographic Column for mRNA Capture Through Understanding of Base‐Pairing Interactions,” Separation and Purification Technology 304 (2023): 122320.

[11] X. Tian , J. Jin , X. Zhu , et al., “Advances and Perspectives in Oligo(dT) Affinity Chromatography for mRNA Capture: Resins, Ligands and Process Intensification,” Journal of Chromatography A 1784 (2026): 467212. 10.1016/j.chroma.2026.467212 42391994

[12] L. Yuan , “Hybridization Liquid Chromatography‐Mass Spectrometry for Quantitative Bioanalysis of Oligonucleotides: The Upcoming Paradigm Shift,” Journal of Chromatography A 1766 (2026): 466575. 10.1016/j.chroma.2025.466575 41344063

[13] V. Gote , P. K. Bolla , N. Kommineni , et al., “A Comprehensive Review of mRNA Vaccines,” International Journal of Molecular Sciences 24 (2023): 2700. 10.3390/ijms24032700 PMC9917162 36769023

[14] T. A. Goss , M. Bard , and H. W. Jarrett , “High‐Performance Affinity Chromatography of DNA,” Journal of Chromatography 508 (1990): 279–287. 10.1016/s0021-9673(00)91270-6 2370282

[15] T. A. Goss , M. Bard , and H. W. Jarrett , “High‐Performance Affinity Chromatography of Messenger RNA,” Journal of Chromatography 588 (1991): 157–164. 10.1016/0021-9673(91)85017-a 1687820

[16] B. Basiri , J. M. Sutton , S. Hooshfar , C. C. Byrnes , M. M. Murph , and M. G. Bartlett , “Direct Identification of Microribonucleic Acid miR‐451 From Plasma Using Liquid Chromatography Mass Spectrometry,” Journal of Chromatography A 1584 (2019): 97–105. 10.1016/j.chroma.2018.11.029 30502920

[17] R. Bumgarner , “Overview of DNA Microarrays: Types, Applications, and Their Future,” Current Protocols in Molecular Biology 101 (2013): 22.1.1–22.1.11. 10.1002/0471142727.mb2201s101 PMC4011503 23288464

[18] Z. Song , A. Lu , and L. Yuan , “Analyte and Probe Melting Temperature Guided Method Development Strategy for Hybridization LC‐MS/MS Quantification of siRNAs,” Journal of Pharmaceutical and Biomedical Analysis 253 (2025): 116556. 10.1016/j.jpba.2024.116556 39504739

[19] T. Ratilainen , A. Holmén , E. Tuite , et al., “Hybridization of Peptide Nucleic Acid,” Biochemistry 37 (1998): 12331–12342. 10.1021/bi9808722 9724547

[20] M. Gilar , B. Muriithi , Y. Sarisozen , P. Wuthrich , M. Donegan , and K. D. Wyndham , “On‐Column Preparation of High‐Performance Affinity Chromatography Columns,” Analytical Chemistry 98 (2026): 24117–24121. 10.1021/acs.analchem.6c01884 PMC13523552 42489360

[21] M. Gilar , S. Redstone , and A. Gomes , “Impact of Mobile and Stationary Phases on siRNA Duplex Stability in Liquid Chromatography,” Journal of Chromatography A 1733 (2024): 465285. 10.1016/j.chroma.2024.465285 39173502

[22] J. Grinsted , J. Liddell , E. Bouleghlimat , et al., “Purification of Therapeutic & Prophylactic mRNA by Affinity Chromatography,” Cell and Gene Therapy Insights 8 (2022): 335–349.

[23] J. Grinsted , E. Bouleghlimat , S. S. Rosa , M. P. C. Marques , and D. G. Bracewell , “Structure‐Performance Analysis of Oligo(dT) Resin Affinity Chromatography for RNA Separations,” Journal of Chromatography A 1762 (2025): 466382. 10.1016/j.chroma.2025.466382 40974705

[24] M. Gilar and U. D. Neue , “Peak Capacity in Gradient Reversed‐Phase Liquid Chromatography of Biopolymers. Theoretical and Practical Implications for the Separation of Oligonucleotides,” Journal of Chromatography A 1169 (2007): 139–150. 10.1016/j.chroma.2007.09.005 17897658

[25] U. D. Neue , “Theory of Peak Capacity in Gradient Elution,” Journal of Chromatography A 1079 (2005): 153–161. 10.1016/j.chroma.2005.03.008 16038301

[26] M. Gilar , C. Doneanu , and M. M. Gaye , “Liquid Chromatography Methods for Analysis of mRNA Poly(A) Tail Length and Heterogeneity,” Analytical Chemistry 95 (2023): 14308–14316. 10.1021/acs.analchem.3c02552 PMC10535021 37696042

[27] J. Demongeot and C. Fougere , “mRNA COVID‐19 Vaccines‐Facts and Hypotheses on Fragmentation and Encapsulation,” Vaccines 11 (2022): 40. 10.3390/vaccines11010040 PMC9864138 36679885

[28] World Health Organization , Messenger RNA Encoding the Full‐Length SARS‐CoV‐2 Spike Glycoprotein (World Health Organization, 2020).

[29] “New NIST Research Grade Test Material to Support mRNA Therapeutics,” NIST , last modified September 24, 2026, https://www.nist.gov/news‐events/news/2025/07/new‐nist‐research‐grade‐test‐material‐support‐mrna‐therapeutics.

