# Supplementary material for: Affinity Chromatography With Selective Nucleic Acid Hybridization: Direct Coupling With Size Exclusion Chromatography
Source: J Sep Sci. 2026 Oct 17;49(10):e70555. doi: 10.1002/jssc.70555 (PMC13675829; doi:10.1002/jssc.70555)
Supplement: Supplementary file 1 [file JSSC-49-e70555-s001.docx]

**Supporting information**

**Affinity Chromatography with Selective Nucleic Acid Hybridization: Direct Coupling with Size Exclusion Chromatography**

Martin Gilar *, Brittney Racioppo, Beatrice Muriithi, and Kevin Wyndham

*Waters Corporation, 34 Maple St., Milford, MA 01757, USA*

* Corresponding author: Martin Gilar – Waters Corporation, 34 Maple Street, Milford, Massachusetts 01757 – 3696, United States, martin_gilar@waters.com

**Supporting Figures:**

Contents

[**Figure S1:** Affinity column preparation.	2](#_Toc241063245)

[**Figure S2:** SEC analysis of EPO mRNA fractions isolated by dT25 column.	3](#_Toc241063246)

[**Figure S3:** Analysis of polyA tailed EPO mRNA, dT150 oligonucleotide and their duplex.	4](#_Toc241063247)

[**Figure S4:** PolyA tail mRNA analysis with SEC and tandem dT25/SEC methods	5](#_Toc241063248)

# **Figure S1:** Affinity column preparation.

Solution of biotinylated oligonucleotides (2 μL injections, 1 injection/min, a 1 min time gap was inserted after each 10 injections series, oligonucleotide concentration was 0.25 nmol/μL; 0.5 nmol/injection) was injected on 2.1 x 20 mm column packed with streptavidin sorbent. Biotinylated oligonucleotide ligands were immobilized on streptavidin sorbent via biotin-streptavidin interaction. Oligonucleotide breakthrough signal was detected at the point of streptavidin column saturation with the oligonucleotide ligand. Greater than 50% breakthrough signal was used to estimate the amount of immobilized ligand. The > 50% breakthrough of biotinylated dT25 was detected at injection 29; 28 injections x 0.5 nmol = estimated amount of immobilized dT25 ligand. TP53 column capacity was estimated in a similar fashion. Column ligand loading was performed with ACQUITY™ Premier System and mobile phase was 0.1 M sodium phosphate buffer, pH 7.4. Mobile phase flow rate was 0.15 mL/min, column temperature 25 °C. For more details see reference 20 of the manuscript (Gilar M., Muriithi, B., Sarisozen, Y., Wuthrich, P., Donegan, M., Wyndham, K.D., On-column preparation of high-performance affinity chromatography columns. *Anal. Chem.* 2026, *in print*).

# **Figure S2:** SEC analysis of EPO mRNA fractions isolated by dT25 column.

dT25 column was used to isolate EPO mRNA fractions 1 (tailless mRNA) and fraction 2 (polyA tailed intact mRNA with the method described in Figure 1 of the main manuscript. Fractions 1 and 2 were digested with RNase T1 to liberate the polyA tail and analyzed with ACQUITY Premier Protein SEC Column, 250Å, 1.7 µm, 4.6 x 150 mm. Mobile phase was 0.1 M sodium phosphate buffer pH 7.4, flow rate 0.2 mL/min, column temperature 25 °C. Fraction 1 (black chromatogram) did not contain a detectable polyA tail peak, which is present in fraction 2 (red chromatogram). SEC analysis of intact EPO mRNA sample (undigested) is shown for comparison (blue chromatogram). RNase T1, used for mRNA digestion, elutes from SEC column at ~7.8 minutes. Minor species eluting prior polyA tail peak are intact mRNA, mRNA species resistant to digestion (aggregated mRNA) or unknown contaminants. The compounds eluting in Fraction 2 near 4 minutes (prior to main intact mRNA peak) must contain polyA tail, because they are retained by dT25 column.

# **Figure S3:** Analysis of polyA tailed EPO mRNA, dT150 oligonucleotide and their duplex.

A) Retention of EPO mRNA, dT150 oligonucleotide and their duplex on dT25 column. B) retention of the same sample on SEC column. mRNA (polyA tailed fraction) is retained on dT25 column, while dT150 oligonucleotide and mRNA:dT150 hybridized duplex is not. This finding suggests that once the polyA tail is hybridized with a complementary dT150 (added in molar excess), the duplexed mRNA polyA tail is “blocked” and no longer retained on dT25 column. Analysis of the same samples in SEC panel B) shows dT150 oligonucleotide peak eluting near 4.7 min, well resolved from EPO mRNA (3.93 min) and EPO mRNA:dT150 duplex (~4.85 min).

# **Figure S4:** PolyA tail mRNA analysis with SEC and tandem dT25/SEC methods

Five different mRNA samples digested with RNase T1 to liberate polyA tail were analyzed with SEC method (black upper chromatograms) or with tandem dT25/SEC method. Flow through dT25 column fraction resolved by SEC column is shown as red chromatograms. dT25 retained fraction was eluted from the column with 25 μL injection-elution method and directly resolved by SEC column (blue chromatograms). Tandem dT25/SEC method provides cleaner results in scenarios when relatively short polyA tail segments are obstructed by other non-polyA tail mRNA digestion products. PolyA tail length obtained from SEC calibration is denoted in chromatograms. Fluc mRNA (NIST) sample contains two polyA tail sections indicated by arrows.
